# Patient Flow and Multidisciplinary Coordination in Urothelial Carcinoma: Insights from Bulgarian Oncologists

**DOI:** 10.3390/healthcare14142202

**Published:** 2026-07-21

**Authors:** Yoanna Vutova, Adriana Krasteva, Tsvetelina Angelova, Georgi Slavchev, Slaveyko Djambazov, Evgeni Grigorov

**Affiliations:** 1 HTA Ltd., 1404 Sofia, Bulgaria; yoanna@hta.bg (Y.V.); a.krasteva@hta.bg (A.K.); tsveti@hta.bg (T.A.); slavchevg@hta.bg (G.S.); slaveykodjambazov@hta.bg (S.D.); 2 Faculty of Pharmacy, Medical University of Varna, 9002 Varna, Bulgaria

**Keywords:** urothelial carcinoma, multidisciplinary team, patient journey optimization, oncology care coordination, value-based healthcare, outcome measurement

## Abstract

**Objectives:** Timely diagnosis, efficient patient journey, and well-coordinated multidisciplinary care are essential foundations for optimizing treatment outcomes in urothelial carcinoma. This study aims to identify key organizational characteristics and development areas within the patient journey of urothelial carcinoma (UC) in Bulgaria, with a particular focus on the role of multidisciplinary teams (MDTs), referral patterns, and the timeliness of therapeutic decision-making. **Methods:** This study employed a structured survey and in-depth interviews with 30 leading Bulgarian oncologists across different medical facilities specialized in the treatment of urothelial carcinoma. The survey was developed according to international best practices and included seven key domains: patient flow, quality of life, health outcomes, patient perception of satisfaction, symptom impact, unmet needs, and trends and changes in therapy. Quantitative survey data were analyzed using descriptive statistics and cross-tabulations, while qualitative interview data were analyzed using thematic analysis to identify key patterns and challenges. **Results:** Most patients entered the care pathway at early disease stages. Referrals were primarily initiated by urologists (71%), while multidisciplinary team involvement was reported in all participating centers, although organizational practices varied. Treatment was initiated within 1–2 weeks following diagnosis in 69% of cases, and follow-up was predominantly conducted through scheduled clinical visits (81%). Respondents identified coordination between healthcare facilities, reduction in waiting times, and improved access to diagnostic tools as the most important areas of progress and ongoing development within urothelial carcinoma care. **Conclusions:** The findings highlight several areas within the urothelial carcinoma patient journey where coordination can be further strengthened, particularly in relation to MDT engagement, meeting regularity, referral pathways, and follow-up practices. Strengthening multidisciplinary collaboration and implementing integrated care pathways may support further improvements in coordination and consistency of care.

## 1. Introduction

### 1.1. Urothelial Carcinoma (UC): Clinical and Organizational Challenge

Urothelial carcinoma (UC) represents the most common histological subtype of bladder cancer, accounting for approximately 90% of all bladder malignancies worldwide [1]. Bladder cancer remains a major global health burden, with an estimated 570,000 new cases and more than 210,000 deaths annually, placing it among the ten most frequently diagnosed cancers worldwide [2]. The disease predominantly affects older populations and shows a marked male predominance, with incidence rates in men being three to four times higher than in women [3].

The clinical management of UC is complex and depends on accurate staging, timely diagnosis, and coordinated treatment planning. International clinical guidelines emphasize the importance of multidisciplinary team (MDT) involvement, particularly in muscle-invasive and advanced disease, to ensure optimal therapeutic decision-making and continuity of care [4,5]. MDT-based approaches have been associated with improved adherence to evidence-based guidelines, more consistent treatment selection and enhanced coordination across specialties [6].

Timeliness of care represents a critical determinant of outcomes in UC. Delays in diagnosis or treatment initiation may negatively affect survival, especially in patients with higher-stage disease, while also increasing patient distress and healthcare system burden [7]. Consequently, well-structured referral pathways and efficient transitions from diagnosis to treatment initiation are central elements of high-quality and value-based oncology care [8].

Despite clear international recommendations, the real-world implementation of MDT coordination and integrated care pathways varies across healthcare systems. Differences in organizational structures, availability of specialist resources, and levels of interinstitutional coordination contribute to variability in patient flow and care delivery [9]. Understanding how these organizational factors influence multidisciplinary collaboration, referral patterns and timeliness of care is essential for identifying opportunities to optimize UC management and improve patient outcomes [10,11].

### 1.2. Role of Multidisciplinary Teams in Oncology

International evidence consistently demonstrates that MDTs are a fundamental component of high-quality oncology care. Structured MDT discussions support more accurate diagnostic assessment, more consistent application of treatment guidelines, and more coordinated decision-making across specialties. By integrating perspectives from surgery, medical oncology, radiation oncology, imaging, and pathology, MDTs strengthen clinical judgment and contribute to more comprehensive and coherent treatment strategies. Across healthcare systems, MDT models have been associated with improved clinical decision processes and more streamlined care delivery [6,9,12].

The principles of value-based care further reinforce the importance of MDT engagement. Value-based care emphasizes outcomes that matter to patients, efficient use of resources, and transparency in the management of the care pathway. Within this framework, MDTs serve as a mechanism that aligns therapeutic planning with clinical priorities, patient needs, and organizational efficiency [8,9].

Their coordinated structure facilitates clearer role allocation, supports shared decision-making, and helps translate evidence-based recommendations into integrated, patient-centered practice. In this way, MDTs contribute directly to improving the quality, consistency, and continuity of oncology care [6,9,11].

Patient flow optimization is increasingly recognized as a key quality indicator in oncology and is closely connected to the performance of MDT processes. Well-organized multidisciplinary workflows can reduce unnecessary delays, improve transitions between diagnostic and treatment stages, and ensure that patients progress smoothly through the care pathway. By enhancing coordination and communication between clinical departments, MDTs help create more predictable and efficient patient trajectories. As a result, MDT functioning represents not only a clinical decision-making structure but also an essential organizational tool for strengthening the overall quality and performance of oncology services [6,11,12].

### 1.3. Healthcare Context in Bulgaria

The healthcare system in Bulgaria provides a well-established structure for managing urothelial carcinoma through specialized hospitals, university centers, and comprehensive oncology facilities. The availability of multidisciplinary capabilities forms a strong foundation for delivering timely and structured cancer care [4,5].

Challenges arise from fragmented processes and limited coordination between different care settings. These variations across institutions, including differences in MDT organization, follow-up approaches, and information flow, represent important opportunities for strengthening continuity of care. As patients may navigate several facilities along their treatment journey, enhancing communication pathways can further support smooth transitions between diagnostic and therapeutic steps [9,11].

In this context, systematic assessment plays a critical role in driving continuous improvement. Evaluating patient flow, MDT functioning, and referral practices enables the identification of both strong existing elements and areas where optimization can elevate efficiency and coherence. Such structured assessment helps advance more integrated and streamlined approaches to urothelial carcinoma care in Bulgaria.

### 1.4. Study Objectives and Research Questions

The primary objective of this study is to identify key organizational bottlenecks and opportunities for improvement in the management of urothelial carcinoma within the Bulgarian healthcare system. The analysis focuses on three core areas that shape care delivery: the structure and functioning of MDTs, referral patterns between specialists, and the timeliness of diagnostic and therapeutic steps.

To support this objective, the study was guided by the following research questions:

RQ1. How are multidisciplinary team (MDT) processes organized and implemented in urothelial carcinoma care in Bulgaria?
RQ2. What referral patterns characterize the journey of patients with urothelial carcinoma?
RQ3. Which organizational factors influence the timeliness and coordination of diagnostic and therapeutic care?

Addressing these questions provides descriptive insights into current organizational practices and opportunities to strengthen coordination within the patient journey through more structured and efficient care processes.

## 2. Materials and Methods

### 2.1. Study Design

The study was designed as a cross-sectional, descriptive analysis aimed at evaluating organizational aspects of urothelial carcinoma care in Bulgaria. Data were collected at a single time point to capture current practices and identify opportunities for system improvement. Quantitative survey findings were complemented by qualitative insights obtained through semi-structured interviews, which were used to provide contextual interpretation of the observed organizational patterns and challenges.

### 2.2. Participants and Sampling

The study targeted 30 leading oncology specialists in Bulgaria who are directly involved in the diagnosis, treatment, and follow-up of patients with urothelial carcinoma. The sampling strategy focused on experienced clinicians with active clinical practice in UC, ensuring that their perspectives reflected real-world organizational processes and current standards of care. This ensured that all respondents had comprehensive insights into patient journey optimization, multidisciplinary coordination, referral dynamics, and care timeliness. A purposive sampling approach was used to recruit specialists with substantial experience in the diagnosis, treatment, and follow-up of patients with urothelial carcinoma. The sample was intentionally designed to include clinicians from leading oncology centers across different regions of Bulgaria in order to capture a broad range of organizational practices and expert perspectives relevant to UC care. The sample size was considered appropriate for obtaining a broad range of expert perspectives from the principal oncology centers involved in urothelial carcinoma care in Bulgaria.

Inclusion criteria required participants to: (1) be actively practicing oncologists with direct involvement in UC management, and (2) be affiliated with recognized oncology centers in Bulgaria. Specialists were identified through professional networks and national oncology associations, ensuring representation from both academic medical centers and community-based oncology practices. Exclusion criteria included clinicians without direct involvement in urothelial carcinoma management, healthcare professionals not actively engaged in clinical practice, and specialists affiliated with institutions that do not routinely provide oncology care for patients with urothelial carcinoma.

The sample included specialists working in well-established medical facilities from multiple Bulgarian regions, enabling the study to capture geographic variability in organizational structures, MDT engagement, and patient flow processes. This broad distribution across different oncology centers strengthened the representativeness of the findings and provided a well-rounded understanding of the key challenges and improvement opportunities within the national context.

### 2.3. Survey Instrument Development

The survey instrument was developed to provide a comprehensive and structured evaluation of organizational processes related to UC care in Bulgaria. Its design followed internationally recognized best practices and relied on validated assessment principles to ensure clarity, consistency, and relevance. The content was informed by the published literature on multidisciplinary cancer care, patient pathway evaluation, healthcare service organization, and value-based healthcare principles. The survey was organized into seven key domains, each capturing an essential dimension of the patient journey:

1. Patient Flow—examining referral pathways, timeliness of diagnostic steps, and transitions between care settings;
2. Quality of Life—addressing factors influencing patients’ daily functioning and well-being;
3. Health Outcomes—exploring perceptions of treatment effectiveness and clinical progress;
4. Patient Satisfaction—capturing patient experience and perceived quality of care;
5. Symptom Impact—assessing how disease symptoms affect functionality and daily routines;
6. Unmet Needs—identifying gaps in support, communication, or service availability;
7. Therapy Trends—reviewing current practices, therapeutic sequencing, and decision-making patterns.

Particular attention was given to aligning the instrument with real-world processes relevant to Bulgarian oncology centers, enabling meaningful interpretation of the results.

A pilot testing phase was conducted with oncology specialists to evaluate clarity, relevance, and response burden. Feedback from this process informed minor refinements in wording, question sequencing, and questionnaire structure. This iterative process ensured that the final instrument was both comprehensive and practical, supporting reliable data collection and facilitating detailed insight into the organization of urothelial carcinoma care in Bulgaria.

### 2.4. Data Collection Procedures

The target population consisted of oncologists actively involved in the management of urothelial carcinoma in Bulgaria. Data were collected using a structured survey complemented by qualitative input, enabling assessment of professional practice patterns, organizational processes and care coordination. Survey-based methodologies are widely applied to evaluate healthcare delivery and system-level performance [11]. Data collection was conducted using anonymous paper-based questionnaires completed by participating clinicians. The questionnaire was developed using standardized sources and frameworks commonly applied in international oncology and healthcare research.

The instrument included items covering patient flow, MDT practices, referral dynamics, and timeliness of care, allowing for the collection of comparable and comprehensive responses.

In addition to the survey, in-depth semi-structured interviews were conducted with all 30 participating clinicians. The interviews were conducted in Bulgarian using a predefined interview guide and enabled deeper exploration of perspectives related to coordination challenges, workflow variability, and opportunities for improving the patient pathway. The semi-structured format allowed experts to elaborate beyond predefined survey items, providing rich contextual details that complemented the quantitative data. Interviews were not audio-recorded and typically lasted between 20 and 30 min, depending on the individual participant and discussion dynamics.

Data collection followed the predefined project timeline: surveys were conducted from December 2024 to February 2025 (3 months), followed by data processing, qualitative transcription, analytical review, and synthesis from February to March 2025. Participants were purposively selected oncology specialists from four hospital institutions across Bulgaria, ensuring representation of diverse organizational settings and clinical perspectives relevant to urothelial carcinoma care. All invited specialists participated in both the survey and interview components, resulting in a 100% response and completion rate.

### 2.5. Data Analysis

Before conducting the main analysis, the research team applied a structured, multi-step analytical approach to ensure consistency and reliability across both quantitative and qualitative data.

Quantitative Analysis: Descriptive statistics were calculated for all survey items, including frequencies, percentages, means, and standard deviations where appropriate. Data were analyzed using Microsoft Excel. Cross-tabulations were performed to examine relationships between variables such as disease stage, gender, and treatment patterns. No inferential statistical tests were performed, as the study was designed to provide descriptive insights into organizational practices and care pathways rather than statistical inference.

Qualitative Analysis: Interview transcripts were analyzed using thematic analysis following Braun and Clarke’s framework. Two independent researchers coded the transcripts, and themes were identified through iterative review and discussion. An inductive coding approach was applied, allowing themes to emerge from the data rather than being predefined. Coding differences were discussed between the researchers and resolved through consensus. Key themes were organized around coordination challenges, workflow processes, and improvement opportunities.

Methodological Triangulation: Quantitative survey findings were systematically compared with qualitative interview insights to identify convergent and divergent patterns. This triangulation strengthened the validity of findings by providing multiple perspectives on organizational processes. Convergent findings were used to strengthen interpretation of the results, while divergent findings were examined to identify alternative perspectives and contextual factors influencing organizational practices.

The analytical approach ensured that the survey responses and interview feedback were examined in a complementary manner.

### 2.6. Ethical Considerations

The study was reviewed by the Ethics Committee of Medical Center “Kardiohelp” (Decision No. 1/2024, 30 September 2024). The Committee determined that the study constitutes non-interventional research involving healthcare professionals only and complies with the applicable ethical requirements. The study was conducted in accordance with the Declaration of Helsinki of 1975, as revised in 2008. All participating clinicians provided informed consent prior to completing the survey and interviews. Data collection procedures ensured full anonymity, and no personal or sensitive information regarding patients or respondents was accessible at any stage. The research team conducting the analysis worked exclusively with aggregated, de-identified datasets, guaranteeing strict confidentiality and compliance with established ethical standards for non-interventional organizational research.

## 3. Results

### 3.1. Patient Demographics and Disease Staging

**Figure 1:**
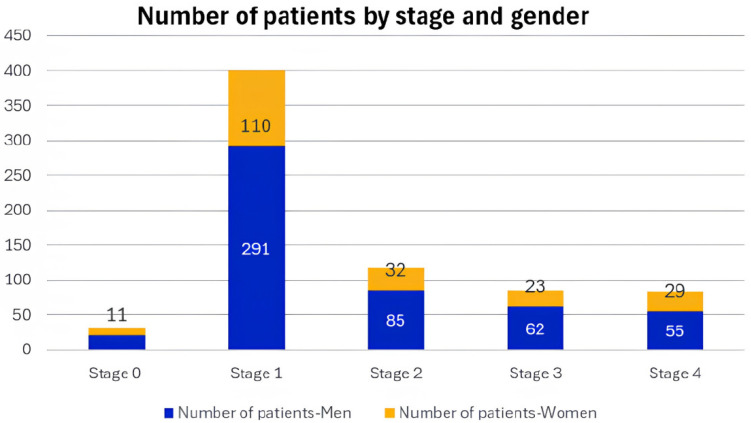
Number of Patients by Urothelial Carcinoma Stage and Gender.

The analysis included an aggregated cohort of 702 patients with urothelial carcinoma managed across the participating centers. As shown in Figure 1, Stage I represents the most frequently reported stage at diagnosis, accounting for the largest proportion of cases within the cohort.

Patient data were derived from actual patients managed by the participating clinicians within their respective institutions during routine clinical practice. Aggregated patient data were used to provide an overall overview of disease stage distribution and treatment patterns across the participating centers.

Earlier-stage disease demonstrates consistent gender patterns. Stage 0 cases are predominantly diagnosed in men, while the distribution in Stage II appears more balanced between male and female patients. In contrast, Stages III and IV again display a higher proportion of male patients, although the difference between sexes is less pronounced than in Stage I. Across all disease stages, male patients constitute the majority of diagnosed cases. Gender distributions across disease stages are presented descriptively, and no inferential statistical comparisons were performed.

In terms of overall distribution, patient numbers decrease progressively with advancing disease stage, indicating that fewer cases are diagnosed at later stages compared with early disease. This pattern highlights that most patients enter the oncology care pathway at an early point in the disease course.

**Figure 2:**
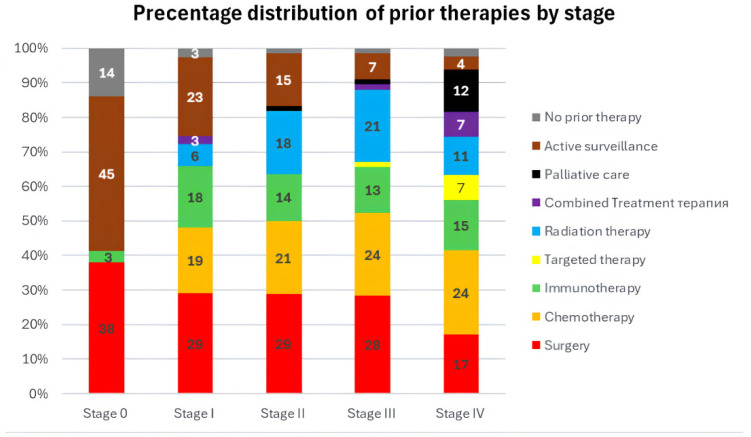
Percentage distribution of prior therapies by disease stage. Note: Chemotherapy and surgery remain the main treatment approaches across all stages.

Patterns of prior therapy differ markedly by stage, as illustrated in Figure 2. Patients diagnosed at earlier stages are most commonly managed with active surveillance, endoscopic surgical procedures and localized interventions, consistent with organ-preserving strategies. As disease stage advances, treatment approaches shift toward more intensive and systemic modalities, including chemotherapy, radiotherapy, combined treatment strategies, and immunotherapy, particularly in Stages III and IV. Across all stages, chemotherapy and surgery remain central components of management, with treatment intensity increasing alongside disease progression.

Overall, the findings demonstrate a patient population dominated by early-stage disease, stable gender differences across stages and stage-appropriate variations in therapeutic approaches.

### 3.2. Referral Patterns and Risk Factor Profile

Referral pathways to oncology services are detailed in Supplementary File S1. Urologists represented the primary referral source (71%), reflecting their central role in the evaluation of urinary symptoms, diagnostic procedures, and early disease management. General practitioners contributed 17% of referrals, highlighting the involvement of primary care in early recognition and initiation of specialist assessment. Diagnostic imaging specialists accounted for 11% of referrals, demonstrating the role of radiological findings in identifying cases, including those detected outside classical urological presentation. The reported percentages reflect clinician-reported assessments of the most common referral sources encountered in routine clinical practice and were rounded to whole numbers for reporting purposes. Therefore, totals may not equal exactly 100%.

The risk factor profile of the patient population is presented in Supplementary File S2. The reported percentages reflect clinicians’ assessments of the relative importance of different risk factors in urothelial carcinoma within their clinical practice and do not represent patient-level prevalence estimates. Tobacco exposure was the most frequently reported risk factor (29%), followed by personal or family history of urothelial malignancy (17%), age ≥ 55 years (15%), and male sex (15%). Chronic inflammatory conditions of the bladder and occupational chemical exposure each accounted for 12% of reported risk factors. This multifactorial risk profile is consistent with established clinical characteristics of urothelial carcinoma and highlights the importance of smoking cessation initiatives and occupational health surveillance in primary prevention strategies.

### 3.3. Multidisciplinary Team Structure and Function

Unless otherwise specified, percentages reported in this section represent responses from participating oncologists and should not be interpreted as percentages of centers.

MDT meeting frequency as reported by participating oncologists varied across institutions (Supplementary File S3). Weekly meetings were reported by 31% of centers, with an additional 31% conducting twice-weekly discussions, supporting timely review of newly diagnosed and ongoing cases. Daily MDT meetings were held by 15% of centers, typically reflecting higher patient volumes and established multidisciplinary capacity. In contrast, 19% of centers reported infrequent or absent MDT meetings, while 4% organized discussions on a case-by-case basis for selected clinical scenarios.

MDT composition showed considerable variation across institutions (Supplementary File S4). Full multidisciplinary structures, including urologists, medical oncologists, radiation oncologists, imaging specialists, and pathologists, were present in 52% of centers. Incomplete MDT configurations, with varying combinations of four specialties, were reported by 36% of centers (12% + 8% + 8% + 8%). The remaining 12% of centers operated with three or fewer specialties represented in their committees.

Leadership roles within MDTs (data from clinician interviews) were most frequently assumed by medical oncologists, while leadership by urologists, radiologists, and pathologists was less commonly reported. Leadership roles within MDTs, based on clinician-reported assessments, were most frequently assumed by medical oncologists (52%), followed by radiologists (17%), urologists (15%), and pathologists (15%) (Supplementary File S4). These findings indicate that medical oncologists play a central coordinating role in multidisciplinary treatment planning and decision-making. Despite this variability in composition and leadership, decision-making processes within MDTs were predominantly collaborative. Treatment decisions were generally reached through consensus among participating specialists or guided by established treatment standards, with no centers reporting reliance on majority voting mechanisms.

### 3.4. Follow-Up Care and Monitoring Practices

Follow-up scheduling for patients with urothelial carcinoma shows notable variation (Supplementary File S5). The most common interval is every three months (43%), reflecting standard post-treatment monitoring practices. Individualized follow-up schedules, adjusted based on disease status and treatment response, account for 31% of cases. Six-monthly reviews are used for 16% of patients, while 5% are monitored monthly and 5% weekly, depending on clinical needs. This distribution indicates a structured, yet flexible approach designed to match follow-up intensity with patient condition.

Monitoring methods after primary treatment (Supplementary File S6) reflect responses from participating oncologists regarding the monitoring approaches most commonly used in routine clinical practice. Percentages were rounded to whole numbers for reporting purposes and therefore may not total exactly 100%. Specialized imaging studies (30%) and physical examination in medical facilities (27%) were the most frequently reported monitoring approaches. Laboratory studies, including blood tests and biomarkers, are used in 19% of follow-up assessments. Combined monitoring approaches are employed in 10% of cases. Telemedicine consultations account for only 6% of follow-up contacts, while patient self-assessment tools (questionnaires or patient reports) represent just 6% of monitoring methods. These patterns illustrate a predominantly clinician-driven and facility-based follow-up model, with limited integration of remote monitoring or patient-reported outcome measures.

### 3.5. Patient Pathway Effectiveness Assessment

Most participating oncologists considered the current system for managing urothelial carcinoma in Bulgaria effective, with 64% rating it as somewhat effective and 32% as very effective. Only 4% reported a neutral assessment, while no respondents rated the system as very ineffective. These findings indicate generally positive perceptions regarding the performance of diagnostic, treatment, and follow-up processes.

Recent developments over the past year demonstrate meaningful progress (Supplementary File S7). The most significant improvement was reported in coordination between healthcare facilities (33%), followed by reduced waiting times (29%) and better access to diagnostic tools (27%). Enhanced patient education and awareness accounted for 12% of reported improvements. Respondents were allowed to select multiple improvement areas; therefore, the reported percentages may exceed 100% when summed.

These advancements collectively indicate a positive trajectory in system performance and reflect ongoing efforts to streamline patient pathways and elevate the quality of urothelial carcinoma care.

### 3.6. Quality of Life, Patient Satisfaction, Symptom Impact, and Unmet Needs

The survey also assessed quality of life, patient satisfaction, symptom impact, and patient support needs as important dimensions of the patient journey in urothelial carcinoma care.

Quality of life was routinely assessed by all respondents, with 80% reporting that it was always evaluated and 20% indicating frequent assessment. Patient interviews (46%) and clinical observations (41%) were the most commonly used methods, while standardized questionnaires were used less frequently (14%). Physical symptoms were identified as the main factor influencing quality of life (38%), followed by psychological well-being (28%).

Patient satisfaction was most commonly assessed through patient interviews (65%) and surveys (26%). The most frequently reported areas of patient feedback included side-effect management (36%), communication with healthcare facilities (27%), treatment effectiveness (20%), and overall care experience (14%). Comprehensive treatment planning (32%) and improved communication (27%) were identified as key approaches for enhancing patient satisfaction.

Symptoms were reported to have a considerable impact on patients’ daily lives. Emotional and mental distress represented the most frequently reported consequence (29%), followed by physical pain and discomfort (25%) and interference with daily activities (22%). Symptoms were also reported to affect work and social participation (14%).

Respondents highlighted several opportunities to further strengthen patient support, including management of treatment-related side effects (32%), access to clinical trials (27%), effective treatment options for advanced disease (22%), and psychological support services (19%). Elderly patients (33%), socioeconomically disadvantaged individuals (28%), and patients with comorbidities (22%) were identified as groups that may particularly benefit from additional support. Collaboration between healthcare facilities (33%), integration of new therapies (26%), and patient education and support (23%) were identified as important areas for continued improvement.

### 3.7. Priority Areas for Improvement

The evaluation of current practices highlights several organizational development areas that could further enhance coordination and consistency in urothelial carcinoma care. These areas, together with the corresponding priority actions, are summarized in Table 1, providing a clear and structured overview of opportunities for strengthening system performance. Table 1 summarizes the most frequently identified organizational shortcomings and improvement priorities emerging from the combined interpretation of survey responses and qualitative interview findings. The items are presented as thematic categories and do not represent direct one-to-one relationships between individual shortcomings and specific improvement priorities.

These aligned development areas and priority actions illustrate a clear and constructive pathway for enhancing UC care. Improvements focused on coordination, diagnostic standardization, and multidisciplinary engagement would support more consistent patient experiences and strengthen overall system performance.

## 4. Discussion

### 4.1. Principal Findings

The findings from this study offer a clear overview of how urothelial carcinoma care is organized and delivered across Bulgarian oncology centers. A notable strength is the early detection profile, with a large proportion of patients being diagnosed at Stage I, reflecting effective initial assessment and timely referral—most often initiated by urologists. The observed patient distribution across disease stages reflects ongoing diagnostic activity and engagement with specialist services within the participating oncology centers.

Multidisciplinary collaboration also emerged as a positive component of current practice. Many institutions conduct MDT meetings on a regular weekly or biweekly schedule, and over half report participation from all key specialties, enabling well-rounded clinical evaluation. Treatment decisions are largely made through consensus or adherence to established standards, supporting consistency and coordination across care settings. Leadership within MDTs is most commonly provided by medical oncologists, indicating active involvement from specialists responsible for longitudinal management.

Despite these strengths, the study also highlights areas where further development could enhance system performance. Nearly one-fifth of centers (19%) report infrequent or absent MDT meetings, and 48% operate with incomplete multidisciplinary structures. These findings point to opportunities for more uniform multidisciplinary integration across the healthcare system. Differences in follow-up organization and treatment timeliness similarly indicate room for process optimization.

Overall, the results describe a system with solid foundations and clear potential for further strengthening multidisciplinary coordination and continuity of care.

### 4.2. Multidisciplinary Coordination: Strengths and Opportunities for Improvement

The MDT data (Supplementary Files S3 and S4) illustrate an overall positive environment for multidisciplinary coordination, with many centers conducting regular tumor board meetings and using consensus-driven decision-making [12]. These practices indicate well-established collaborative structures and a clear commitment to interdisciplinary evaluation.

At the same time, the visual data show notable variation across institutions. Differences in meeting frequency, incomplete MDT composition and concentrated leadership roles suggest that not all centers benefit equally from the full multidisciplinary model [13]. Occasional oncology-only committees further narrow the expertise available during case discussions.

Together, these patterns point to clear opportunities for strengthening MDT practice. Increasing meeting regularity, ensuring participation from all relevant specialists and broadening leadership involvement would help align institutions with the strong models already present elsewhere and promote more integrated, comprehensive care [14].

The implications for patient outcomes are meaningful: centers with consistent MDT engagement and full specialist representation are better positioned to deliver coordinated, guideline-aligned treatment. Improving uniformity across institutions would reinforce decision-making quality and support more reliable clinical pathways for patients with urothelial carcinoma [6].

### 4.3. Timeliness of Care: Progress and Persistent Delays

Data on care timeframes were collected through structured clinician interviews and represent expert assessment of typical patient pathways in their respective institutions. The clinician-reported diagnostic timelines suggest that a substantial proportion of patients receive confirmation of urothelial carcinoma within relatively short timeframes. These findings indicate opportunities to maintain timely diagnostic pathways while continuing efforts to improve early symptom recognition and referral processes. Greater awareness among the public and primary care providers could help further shorten the diagnostic interval.

The transition from diagnosis to treatment initiation also demonstrates opportunities for effective coordination between diagnostic confirmation, MDT decision-making, and treatment scheduling. The reported treatment initiation timelines suggest relatively short intervals between diagnosis and therapy for many patients. Such timely progression may support continuity of care during the early stages of the patient journey.

A smaller but important subgroup experiences delays beyond two weeks. These may stem from scheduling limitations, administrative processes or case complexities requiring additional review. Prolonged waiting can impact clinical outcomes and increase anxiety, underscoring the value of addressing contributing factors to achieve more uniform timeliness across institutions.

The reported diagnostic and treatment initiation timelines indicate opportunities to maintain timely care delivery while continuing efforts to reduce variation across institutions and patient groups. Continued optimization of referral pathways, diagnostic processes, and treatment coordination may further improve consistency across the care continuum.

Overall, the reported timeframes provide insights into the organization of diagnostic and treatment pathways while highlighting opportunities to enhance early recognition, streamline administrative processes, and reinforce coordination within urothelial carcinoma care.

### 4.4. Follow-Up and Monitoring: Traditional Approaches and Digital Opportunities

Follow-up care for patients with urothelial carcinoma remains centered on regularly scheduled in-person visits (physical examination: 27%, imaging: 30%), which allow for direct clinical assessment and timely adjustment of management plans. While this model ensures reliable oversight, it is resource-intensive and may create logistical burden for patients who require frequent travel or have limited flexibility. These factors highlight the value of exploring complementary approaches that could ease pressure on both patients and healthcare facilities.

Digital health methods, including telemedicine and remote symptom monitoring, are used infrequently (telemedicine: 6%, patient self-assessment tools: 6%) despite their potential benefits [15]. Limited adoption reflects missed opportunities to support early identification of symptom changes and to provide more accessible follow-up options for patients, particularly those living farther from oncology centers [16]. Patient-reported outcome tools could further enhance monitoring by offering structured, real-time insights into clinical status, yet they remain underutilized [17]. Barriers such as workflow integration, infrastructure variation and clinician familiarity contribute to the slow uptake of these approaches.

International experience demonstrates that combining traditional follow-up with digital tools can improve patient satisfaction, reduce unnecessary visits and optimize resource use [16]. Applying similar strategies could strengthen the follow-up model for urothelial carcinoma by increasing flexibility and responsiveness.

Future improvements may include broader integration of remote monitoring, routine use of patient-reported outcomes and clear protocols that guide when digital or in-person follow-up is most appropriate. Such enhancements would complement existing practices rather than replace them, supporting more efficient and patient-centered care.

### 4.5. Behavioral Dimensions of Care Coordination

The coordination of urothelial carcinoma care is influenced not only by organizational structures but also by behavioral patterns among providers, patients and institutions. On the provider side, differences in MDT participation and team composition reflect varying levels of engagement in shared decision making. In many centers, clinicians demonstrate a collaborative approach supported by regular meetings and guideline-oriented decisions. In others, more limited specialist involvement or individualized decision styles suggest opportunities to strengthen inter-professional communication and consistency in applying established protocols.

Based on clinician-reported observations, patient-related factors may also influence pathway effectiveness. Delays in presentation following symptom onset may partly reflect differences in symptom recognition and healthcare-seeking behavior. Although these aspects were not directly assessed in the present study, participating clinicians identified them as potential contributors to diagnostic delays. In follow-up care, engagement remains largely driven by scheduled in-person visits, with limited use of self-monitoring or digital tools. This pattern highlights the potential value of supporting patients through clear communication, accessible information, and structured follow-up guidance.

At system level, behavioral dimensions appear in how institutions communicate, coordinate and allocate resources. Recent improvements in coordination and waiting times suggest that many centers have adopted practices that reinforce cooperative behaviors. Nevertheless, variation in MDT regularity (19% with infrequent/absent meetings), incomplete team composition (48% of centers), and limited digital monitoring adoption (telemedicine: 6%) indicate that such behaviors are not yet embedded uniformly across all facilities.

These observations point to several opportunities for behavior-focused interventions. Educational initiatives for providers can encourage consistent MDT participation and protocol adherence. Patient education efforts may support earlier presentation and sustained engagement in follow-up. Structural adjustments that simplify communication and clarify shared responsibilities can further promote coordinated behavior across the care continuum.

### 4.6. Alignment with Value-Based Healthcare Principles

Several current practices in urothelial carcinoma care align well with value-based healthcare principles, particularly the use of MDT discussions, timely diagnostic-to-treatment transitions and recent improvements in coordination. These elements support consistent, patient-centered and outcome-oriented care. At the same time, variation in MDT structures, monitoring approaches and digital follow-up use highlights opportunities to strengthen value by improving standardization and expanding more efficient models of care. A clearer focus on outcome measurement, including timeliness, adherence and patient experience, would further reinforce value-based performance [18].

### 4.7. Study Strengths and Limitations

The study demonstrates several key strengths, including a comprehensive evaluation of urothelial carcinoma care across multiple domains and the involvement of 30 leading specialists from multiple Bulgarian regions who contribute informed, practice-based perspectives. The 100% response rate ensures comprehensive representation of current practices. The combined use of quantitative and qualitative data provides a balanced understanding of coordination patterns and pathway performance.

At the same time, several limitations should be noted. The sample size and geographic coverage may not reflect all institutional contexts nationally. Self-reported data introduce the possibility of perceptual bias, and the cross-sectional design limits causal interpretation. Generalizability may also be influenced by structural variations between centers. Additional limitations include reliance on clinician-reported data rather than direct patient or administrative records for some metrics, potential recall bias in retrospective reporting of patient numbers and timeframes, the absence of patient perspectives, which may provide different insights into care quality and coordination, limited data on clinical outcomes, which restricts assessment of the relationship between organizational factors and patient results.

Despite these considerations, the study offers a meaningful overview of current practices and identifies clear directions for further system improvement.

## 5. Conclusions

The study identifies several systemic inefficiencies that influence the consistency of urothelial carcinoma care. Variation in MDT engagement, including irregular meetings and incomplete specialist participation, remains a central concern. Follow-up practices rely primarily on in-person visits, limiting flexibility, while a subset of patients continue to experience delays between diagnosis and treatment initiation.

The findings highlight several areas that may warrant consideration for future service development and quality improvement initiatives. Immediate priorities may include (1) encouraging regular MDT meetings across centers treating urothelial carcinoma, ensuring participation from urologists, medical oncologists, radiation oncologists, imaging specialists, and pathologists, (2) consideration of standardized diagnostic-to-treatment pathways aimed at reducing unnecessary delays in treatment initiation, and (3) development of shared leadership models within MDTs to distribute responsibility and enhance multidisciplinary engagement. Medium-term goals include (1) the expansion of telemedicine capabilities for routine follow-up consultations, particularly for stable patients and those in remote locations, (2) integration of patient-reported outcome measures into standard follow-up protocols to enable proactive symptom monitoring and (3) creation of integrated electronic care pathways connecting primary care, urology, and oncology services. Long-term system improvements may include:

(1) establishing national benchmarking standards for MDT performance, treatment timeliness, and follow-up quality;
(2) developing telemonitoring infrastructure to support hybrid in-person and remote follow-up models; and
(3) implementing continuous quality improvement mechanisms based on systematically collected outcome data.

These conclusions highlight opportunities to strengthen coordination, multidisciplinary collaboration, and monitoring practices within the Bulgarian healthcare system. Future research should involve broader participation across additional centers, longitudinal assessment of implementation outcomes, incorporation of patient perspectives, and patient-centered measurement of care quality. Such research would further clarify progress, identify best practices, and guide continued improvement toward more integrated, value-based oncology care.

## Figures and Tables

**Table 1:** Organizational Shortcomings and Priority Areas for System Improvement in UC Care.

Organizational Shortcomings Identified	Specific Improvement Priorities
Patient education deficiencies	Enhancing coordination between facilities
Data coordination challenges	Reducing delays between specialist consultations
Post-treatment monitoring limitations	Improving histopathological evaluation
MDT formalization needs	Ensuring adequate staging before treatment
	Strengthening multidisciplinary collaboration

## Data Availability

The raw data supporting the conclusions of this article will be made available by the authors on request.

## Supplementary Materials

The following supporting information can be downloaded at: https://www.mdpi.com/article/10.3390/healthcare14142202/s1, Supplementary File S1. Referral Pathways for Patients with Urothelial Carcinoma. Supplementary File S2. Risk Factors for Urothelial Carcinoma Reported by Participating Oncologists. Supplementary File S3. Frequency of Multidisciplinary Team (MDT) Meetings. Supplementary File S4. Leadership Roles within Multidisciplinary Teams. Supplementary File S5. Composition of Multidisciplinary Teams in Urothelial Carcinoma Care. Supplementary File S6. Strategies for Improving Patient Satisfaction in Clinical Practice. Supplementary File S7. Assessment of Symptom Changes in Urothelial Carcinoma Patients. Supplementary File S8. Common Areas of Patient Feedback in Urothelial Carcinoma Care. Supplementary File S9. Strategies for Responding to Unmet Needs in Urothelial Carcinoma Care. Supplementary File S10. Specific Patient Groups with Insufficiently Addressed Needs. Supplementary File S11. Quality of Life Assessment in Urothelial Carcinoma: Frequency, Methods, and Key Influencing Factors.

## Abbreviation

UC	Urothelial carcinoma
MDTs	multidisciplinary teams
