# Supplementary figures and images for: Patient Flow and Multidisciplinary Coordination in Urothelial Carcinoma: Insights from Bulgarian Oncologists
Source: Healthcare (Basel). 2026 Jul 21;14(14):2202. doi: 10.3390/healthcare14142202 (PMC13411170; doi:10.3390/healthcare14142202)

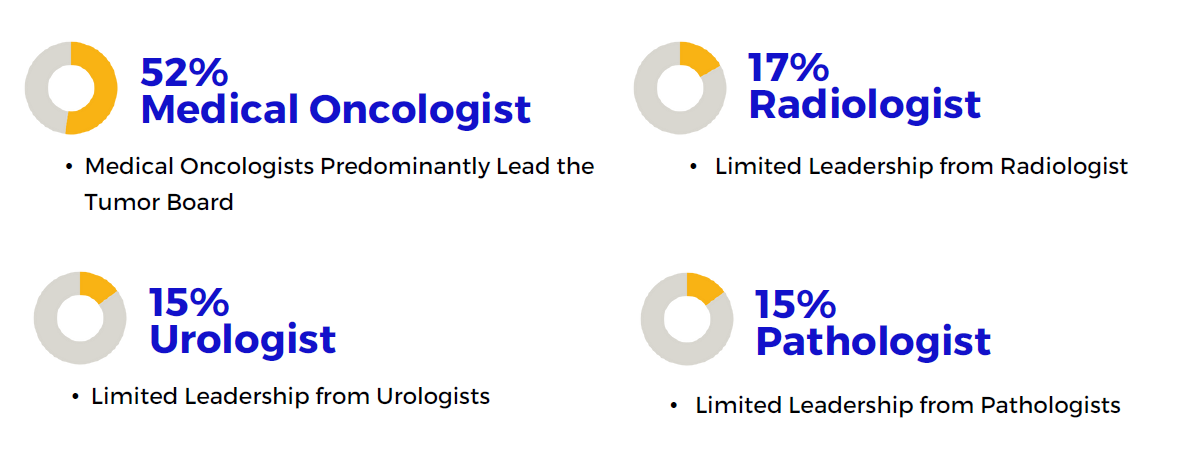

Supplement: Supplementary file 1 [file healthcare-14-02202-s001.zip › Suppl. file S4 (1).png]

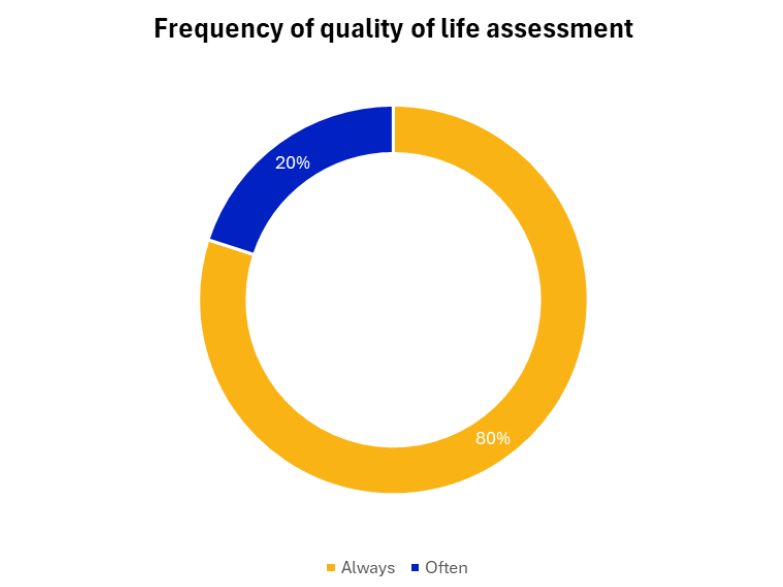

Supplement: Supplementary file 1 [file healthcare-14-02202-s001.zip › Supplementary File S1.png]

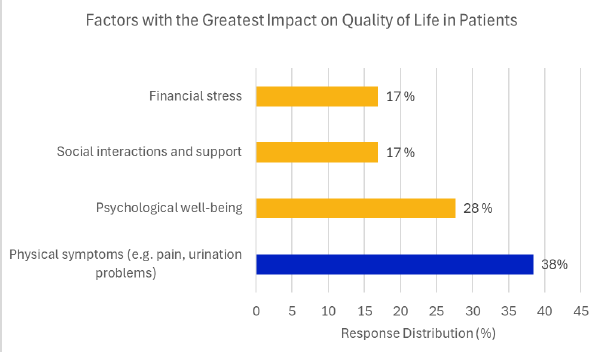

Supplement: Supplementary file 1 [file healthcare-14-02202-s001.zip › Supplementary_File S10.png]

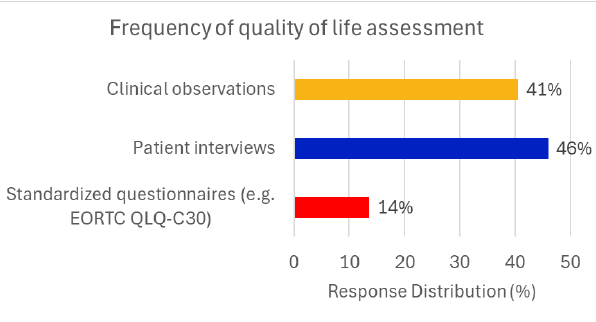

Supplement: Supplementary file 1 [file healthcare-14-02202-s001.zip › Supplementary_File S11.png]

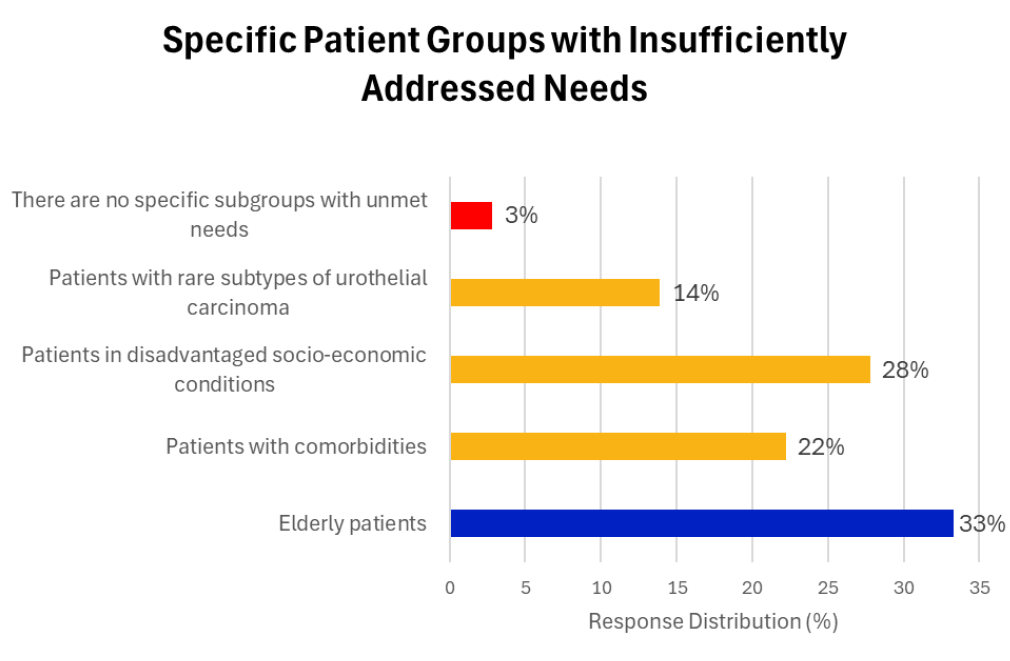

Supplement: Supplementary file 1 [file healthcare-14-02202-s001.zip › Supplementary_File S2.png]

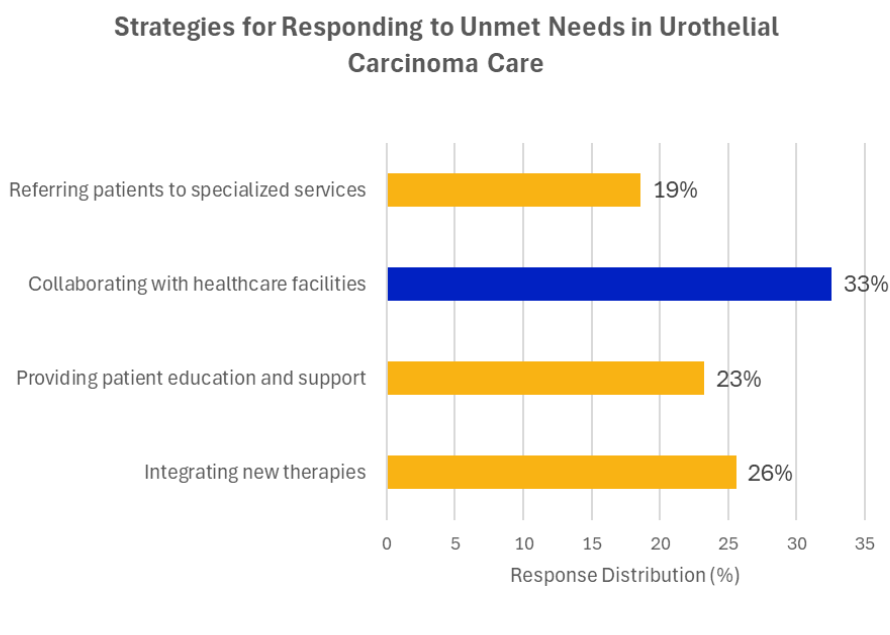

Supplement: Supplementary file 1 [file healthcare-14-02202-s001.zip › Supplementary_File S3.png]

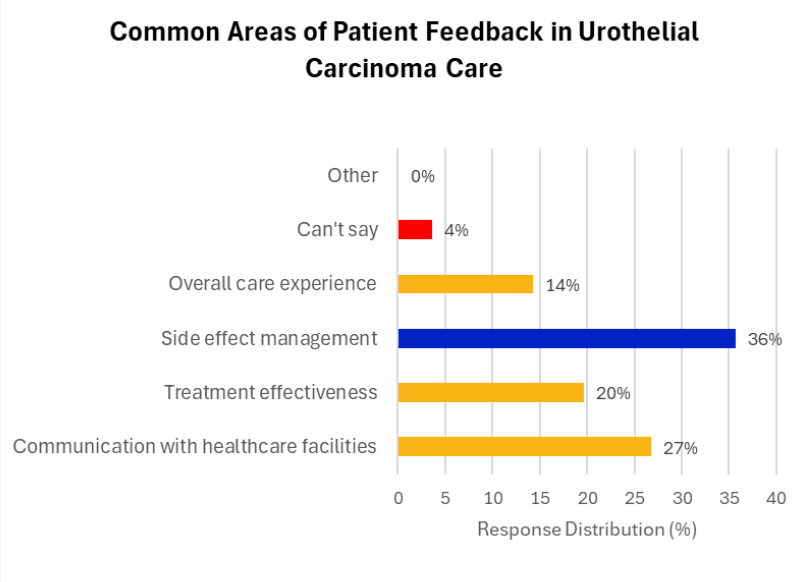

Supplement: Supplementary file 1 [file healthcare-14-02202-s001.zip › Supplementary_File S5.png]

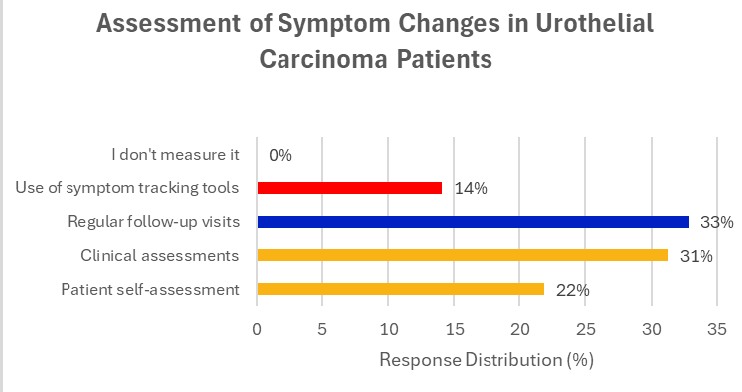

Supplement: Supplementary file 1 [file healthcare-14-02202-s001.zip › Supplementary_File S6.png]

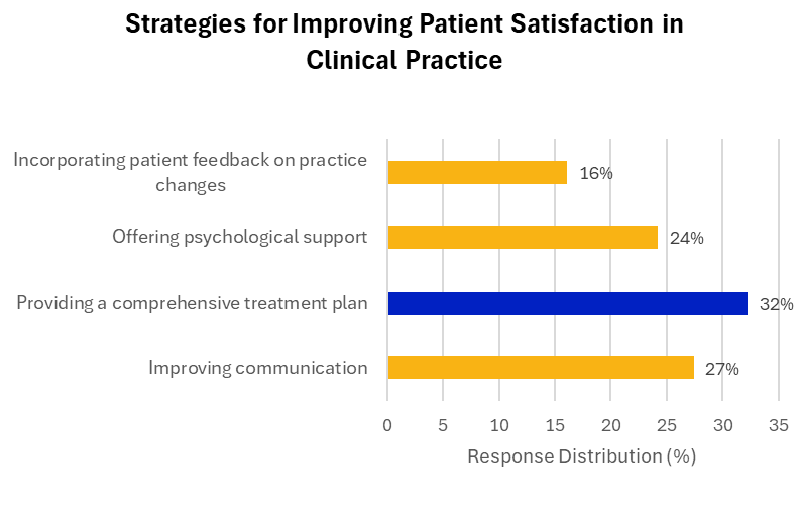

Supplement: Supplementary file 1 [file healthcare-14-02202-s001.zip › Supplementary_File S7.png]

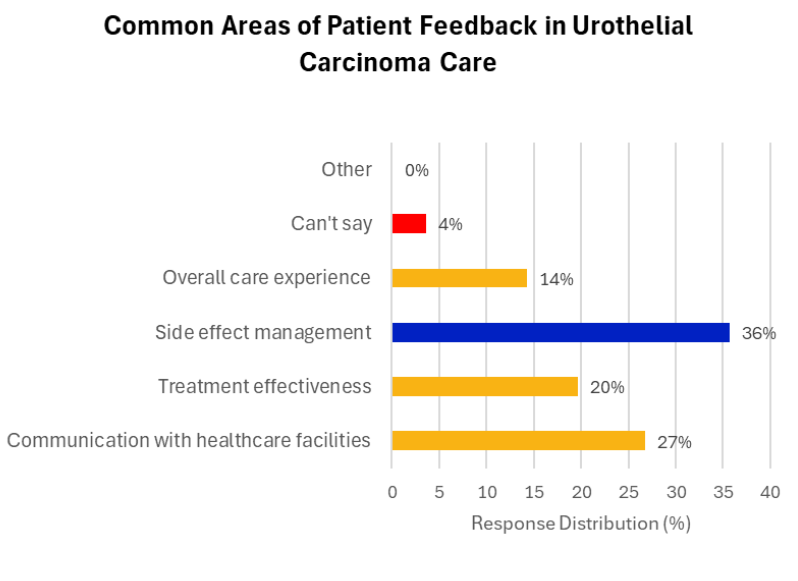

Supplement: Supplementary file 1 [file healthcare-14-02202-s001.zip › Supplementary_File S8.png]

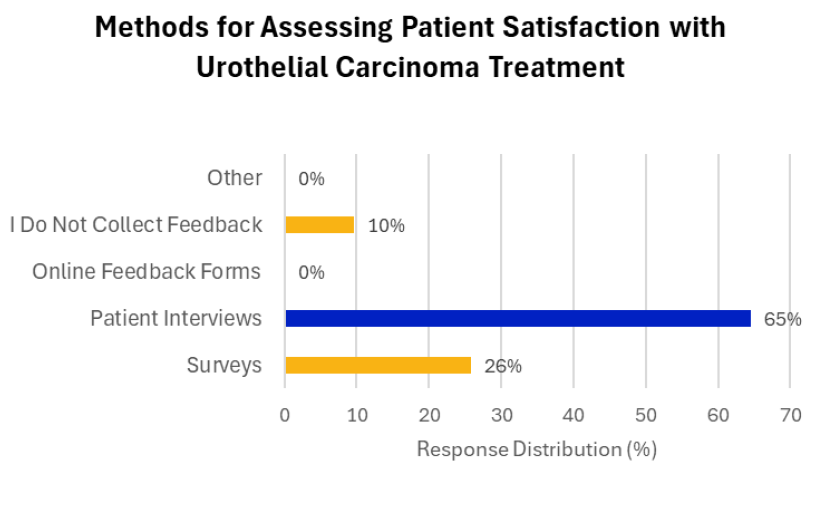

Supplement: Supplementary file 1 [file healthcare-14-02202-s001.zip › Supplementary_File S9.png]
